# Whole-Exome Sequencing Reveals Clinical Potential of Circulating Tumor DNA from Peritoneal Fluid and Plasma in Endometrial Cancer

**DOI:** 10.3390/cancers14102506

**Published:** 2022-05-19

**Authors:** Hye-Yeon Ju, Jung Yoon Ho, Jun Kang, Soo Young Hur, Sejin Kim, Youn Jin Choi, Mi-Ryung Han

**Affiliations:** 1 Division of Life Sciences, College of Life Sciences and Bioengineering, Incheon National University, Incheon 22012, Korea; jhyhy0525@gmail.com; 2 Department of Obstetrics and Gynecology, Seoul St. Mary’s Hospital, College of Medicine, The Catholic University of Korea, Seoul 06591, Korea; hojy2000@catholic.ac.kr (J.Y.H.); hursy@catholic.ac.kr (S.Y.H.); ksally37@gmail.com (S.K.); 3 Cancer Research Institute, College of Medicine, The Catholic University of Korea, Seoul 06591, Korea; 4 Department of Hospital Pathology, Seoul St. Mary’s Hospital, College of Medicine, The Catholic University of Korea, Seoul 06591, Korea; jkang.alien@gmail.com

**Keywords:** endometrial cancer, circulating tumor DNA, plasma, peritoneal fluid, liquid biopsy, heterogeneity, whole-exome sequencing, somatic mutations, copy number alterations, microsatellite instability

## Abstract

**Simple Summary:**

Studies on biomarkers for endometrial cancer using circulating tumor DNA (ctDNA) from liquid biopsy samples are lacking. The aim of this study was to analyze gDNA from tumor tissues and ctDNA derived from peritoneal fluid and plasma samples and determine their mutational concordance via microsatellite instability, copy number alteration, and mutational signature analyses using whole-exome sequencing and P53 immunohistochemistry. ctDNA of two patients with negative cytology presented *TP53* mutations concordant with those in the tissue, and the ctDNA of a patient with positive cytology harbored both *TP53* and *POLE* mutations, although none were detected in the tissue. This study is the first to demonstrate comprehensive genomic concordance between ctDNA from the peritoneal fluid and tumor gDNA in endometrial cancer at the whole-exome level.

**Abstract:**

Endometrial cancer (EC) is the most common type of gynecological cancer. Studies comparing tumor gDNA and ctDNA isolated from the plasma and peritoneal fluid of EC patients are limited. Whole-exome sequencing and P53 immunohistochemistry of 24 paired tissue, plasma, and peritoneal fluid samples from 10 EC patients were performed to analyze somatic mutations, copy number alterations, microsatellite instability, and mutational signatures. Mutations in cancer-related genes (*KMT2C*, *NOTCH2*, *PRKAR1A*, *SDHA*, and *USP6*) and genes related to EC (*ARID1A*, *CTNNB1*, *PIK3CA*, and *PTEN*) were identified with high frequencies among the three samples. *TP53* and *POLE* mutations, which are highly related to the molecular classification of EC, were identified based on several key observations. The ctDNA of two patients with negative peritoneal fluid presented *TP53* mutations concordant with those in tissues. ctDNA from the plasma and peritoneal fluid of a patient with positive cytology harbored both *TP53* and *POLE* mutations, although none were detected in tissues. Additionally, the patient presented with wild type P53 immunohistochemistry, with a focal “high” expression in a “low” wild type background. The tissues and peritoneal fluid of 75% EC patients showed concordant microsatellite instability. Furthermore, we observed strong mutational concordance between the peritoneal fluid and tumors. Our data suggest that the ctDNA from peritoneal fluid might be a suitable biomarker for identifying the mutational landscape of EC and could complement tumor heterogeneity.

## 1. Introduction

Endometrial cancer (EC) is the most common gynecological cancer affecting the lining of the uterus [1]. Previous studies have shown that the molecular classification of EC plays an important role in the formulation of therapeutic strategies [1,2]. The Cancer Genome Atlas (TCGA) classifies EC into four groups—POLE ultramutated, MSI hypermutated, copy number (CN) low, and CN high, which correlate with progression-free survival. However, the methodologies used for classification in TCGA studies are highly complex and expensive to be used in clinical applications; therefore, comparative pragmatic methodologies have been developed [3,4]. Two previous studies performed *TP53* mutational testing/p53 immunohistochemistry (IHC) to determine p53 status as a surrogate for classification of the CN-high TCGA group [5]. This approach demonstrated a risk discriminatory ability comparable to that provided by the European Society of Medical Oncology (ESMO) risk stratification system. The recent European Society of Gynaecological Oncology guidelines suggest the possibility of incorporating different therapeutic options based on molecular classifications, i.e., tumors with (1) *POLE* mutations, (2) *P53*-abnormal, (3) mismatch repair deficiency (MMRd), and (4) non-specific molecular profile (NSMP) [6]. Patients with *POLE*mut tumors have excellent prognoses, those with abnormal p53 tumors have poor prognoses, and those with MMRd or NSMP have intermediate prognoses [6].

Cell-free DNA (cfDNA) molecules were first discovered in the human circulatory system in 1948, and a number of studies have demonstrated the potential use of cfDNA as a noninvasive biomarker for multiple phenotypes of cancer [7]. A small proportion of cfDNA, called circulating tumor DNA (ctDNA), originates from primary sites, metastatic sites, or circulating tumor cells (CTCs). Compared to tissue biopsy, liquid biopsy is a minimally invasive method for the investigation of tumor cells (CTC), circulating tumor DNA (ctDNA), and tumor-derived exosomes [8].

Previous studies have suggested that the ctDNA from peritoneal fluid can provide additional genomic information that cannot be obtained from tumor and plasma ctDNA in various cancers; therefore, ctDNA may be useful in clinical practice [9,10,11]. National Comprehensive Cancer Network (NCCN) guidelines (https://www.nccn.org/; accessed on 10 September 2021) suggest that peritoneal fluid should be included in surgical procedures. Although cytology alone does not affect staging, results should still be obtained, because positive cytology is considered an adverse risk factor. Recent genomic studies of tumor ctDNAs isolated from plasma and peritoneal fluid have been conducted using targeted sequencing approaches. Concomitant *KRAS* and *PIK3CA* mutations among the tumor, plasma, and peritoneal fluid in EC have been reported using targeted sequencing of gDNA derived from tissues and quantitative polymerase chain reaction (PCR) assay for ctDNA derived from the plasma and peritoneal fluid [12]. Another study used a custom panel targeting 30 hot spot mutations in four genes (*CTNNB1*, *KRAS*, *PTEN*, and *PIK3CA*) and found that 33% of EC patients had mutations in the plasma that matched mutations in the tumors [13]. In addition, a previous study suggested that EC plasma ctDNA might detect microsatellite instability (MSI) status [14]. However, to date, genomic studies comparing cfDNA isolated from the plasma and peritoneal fluid in EC patients at the whole-genome or whole-exome level are non-existent. Therefore, it is necessary to investigate the mutational profiles of EC, in addition to well-known EC-related gene mutations, including *TP53*, *PTEN*, *KRAS*, and *PIK3CA*.

In the present study, whole-exome sequencing (WES) of gDNA obtained from the tissues and ctDNA derived from the plasma and peritoneal fluid of 10 patients with EC was performed. Comprehensive mutational profiles, copy number alterations (CNAs), MSI, and mutational signatures of the tissue, plasma, and peritoneal fluid were simultaneously analyzed in order to elucidate mutational concordance. In addition, p53 immunohistochemistry was performed for a more comprehensive study.

## 2. Materials and Methods

### 2.1. Samples

**Figure 1:**
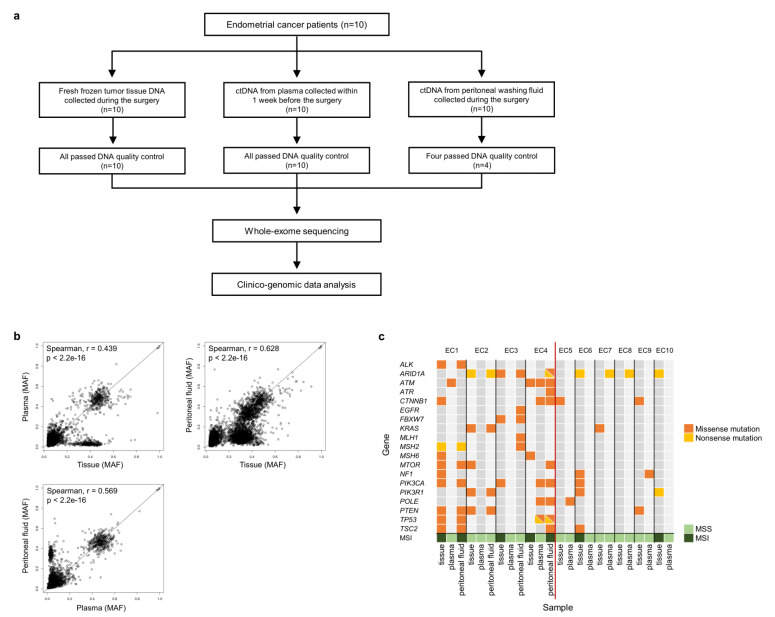
(**a**) Patient flow chart. (**b**) Comparison of mutant allele frequencies of the mutations shared between tissue and plasma, tissue and peritoneal fluid, and plasma and peritoneal fluid. The mutant allele frequencies (MAF) are shown on the *x*-axis and *y*-axis. (**c**) Somatic mutations and microsatellite instability in matched tissue and ctDNA samples using the top 20 EC-related genes. Samples are shown on the *x*-axis, and EC-related genes are listed on the *y*-axis. Nonsynonymous mutations and nonsense mutations are shown in orange and yellow, respectively. The color of MSI status indicates MSS and MSI (MSS, microsatellite stability; MSI, microsatellite instability).

All specimens from the EC patients in this study were obtained with appropriate consent and approval from the Institutional Review Board of Seoul St. Mary’s Hospital, Catholic University of Korea, College of Medicine (KC17TNSI0215 and XC16TISI0014K). Fresh tumor tissues and peritoneal cytological fluid were collected during primary staging surgery. Tumor tissues from 10 EC patients were collected; the size of the tissue from each EC patient was ≥0.5 × 0.5 × 0.5 cm^3^. Peritoneal fluid was collected (~10 ml) from four of the 10 patients. Ten milliliters of blood were collected from each patient before surgery. Thus, a total of 10 tumor tissues, 10 blood samples, 4 peritoneal fluid samples, and 10 buffy coat samples for matched controls were obtained from 10 patients with EC (Figure 1a).

### 2.2. Preparation of ctDNA and gDNA

Using gDNA obtained from the tissue and ctDNA obtained from the plasma and peritoneal fluid, exome capture was performed using the Agilent SureSelect Target Enrichment protocol for the Illumina paired-end sequencing library (Version C2, December 2018). For all cases, the Agilent SureSelect Human All Exon V6 probe set (Agilent Technologies, Santa Clara, CA, USA) was used. After washing and amplification of the captured DNA, the final purified product was quantified using the KAPA Library Quantification kit (Roche Sequencing Solutions, Pleasanton, CA, USA), and its quality was assessed using TapeStation DNA screentape D1000 (Agilent Technologies, Santa Clara, CA, USA). Sequencing was performed on Novaseq6000 platform (Illumina, San Diego, CA, USA).

### 2.3. WES and Somatic Mutations

The adapter sequences were removed using Agilent SurecallTrimmer v4.0.1, and trimmed reads were mapped to the human reference genome (GRCh37/hg19) using BWA-MEM v0.7.12 [15]. Poorly mapped reads with mapping quality below 20 and duplicated reads were discarded using Samtools v1.3.1 [16] and LocatIt v4.0.1 (Agilent Technologies, Santa Clara, CA, USA), respectively. The base quality of the duplicated reads was recalibrated using the Genome Analysis Toolkit (GATK v3.7) and BaseRecalibrator. Somatic mutations and indels were detected using MuTect2 [17]. For functional annotation of each variant in the coding region, ANNOVAR (annotated variation) [18] and SnpEff v4.3t [19] were used. A frequency of 3% was applied for gDNA and 0.1% for ctDNA to assess mutations, as most ctDNAs originate from dying noncancerous cells. Germline variants were eliminated when the minor allele frequency was >1% in the Genome Aggregation Database (East Asian) and the Korean Variant Archive (KOVA) [20]. PolyPhen-2, PROVEAN, and SIFT were used to predict the effects of amino acid substitutions on protein functions and structures [21,22,23].

### 2.4. Mutational Signature Analysis

Somatic mutational signatures were estimated using SigProfilerMatrixGenerator v1.1.20 (Alexandrov Lab, San Diego, CA, USA) and SigProfilerExtractor v1.0.17 (Alexandrov Lab, San Diego, CA, USA), wherein an optimal set of mutational signatures was deciphered based on a non-negative matrix factorization (NMF) algorithm [24]. The algorithm uses multiple NMF iterations (in most cases, 1024), and in each iteration, SigProfilerExtraction minimizes a generalized Kullback–Leibler divergence constrained for nonnegativity [25]. To analyze the mutational signatures of the tissue, plasma, and peritoneal fluid samples, similarities were calculated using the Catalog of Somatic Mutations in Cancer (COSMIC) mutational signatures v3.1, which includes single-base-substitution (SBS), doublet-base-substitution (DBS), and small insertion-and-deletion (ID) signatures [26]. Similarities between mutational signatures were calculated using cosine correlation similarity ranging from 0 (completely non-identical) to 1 (completely identical) [27].

### 2.5. Copy Number Alteration

Copy number calling was performed on all paired tumor-normal sequencing data using CNVkit v0.9.7 [28]. CNAs were analyzed using two categories: copy number gain (CN gain:3–7 copies) and copy number loss (CN loss:0–1 copies) [29]. The (CBS) algorithm, a modification of binary segmentation to divide regions with equal copy numbers, was applied to identify genomic aberrations [30]. After segmentation, the genomic identification of significant targets in cancer (GISTIC) algorithm was applied to compare the gene-level CNAs. To determine the significant amplification and deletion regions, the significance threshold for the *q*-values was set to 0.25 [31]. We grouped the tissue, plasma, and peritoneal fluid samples separately and analyzed the recurrent regions of CNAs for each group. CNTools was used to convert segment data into a gene by sample matrix, and a total of 666 cancer-related genes from the Cancer Gene Census (http://cancer.sanger.ac.uk/census; accessed on 10 August 2021) and genes previously reported in EC were used [1,12,13,32,33,34]. Copy number alteration data were hierarchically clustered using Manhattan distance and Ward’s method.

### 2.6. Microsatellite Instability

The MSI status was estimated using the MANTIS algorithm, which was developed to detect MSI with high sensitivity and specificity across a variety of cancer types [35]. Euclidian distance was used as a metric to determine the MSI status of the samples. Samples with values greater than or equal to the threshold were classified as demonstrating MSI, and the other samples were classified as microsatellite stable (MSS). Samples were categorized into four molecular groups based on the MSI status, DNA polymerase epsilon (*POLE*) exonuclease domain mutations (EDM), and copy number alteration patterns [1,36]. MSI is a marker of mismatch repair deficiency (MMRd), and testing for MMRd and MSI has been reported to be relevant. MSI testing is often used to predict MMR protein status [37,38].

### 2.7. Immunohistochemistry

Formalin-fixed, paraffin-embedded blocks were cut into 4 μm-thick sections. Immunohistochemical staining for p53 was performed using an automated Ventana BenchmarkXT slide stainer (Ventana, Tucson, AZ, USA) with a primary antibody against p53 (pre-diluted, DO-7, Roche Diagnostics, IN, USA). The staining patterns were classified as follows: (1) overexpression, if at least 80% of the tumor cell nuclei showed diffuse strong nuclear staining; (2) complete absence, if the tumor cell nuclei showed no positive nuclei; (3) wild type, if the tumor cells showed staining patterns between overexpression and complete absence; and (4) cytoplasmic, if the tumor cells showed unequivocal cytoplasmic staining. The wild type pattern was interpreted as normal/wild type, and the other patterns were interpreted as abnormal/aberrant/mutation type [39].

## 3. Results

### 3.1. Patients, Samples, and Clinical Data

We analyzed 10 tumor tissues, 10 plasma samples, and four peritoneal fluid samples from 10 patients with EC (Figure 1a). All patients underwent staging surgery; their clinicopathological features are described in Table 1. The age of patients at diagnosis ranged from 45 to 74 years (median, 57 years). The patients were classified into stages I–IV based on the 2009 International Federation of Gynecology and Obstetrics (FIGO) guidelines, with a histopathological diagnosis of endometrioid adenocarcinoma (n = 10). Two (20%) patients were diagnosed with FIGO stage I disease, one (10%) with stage II, six (60%) with stage III, and one (10%) with stage IV disease. Grade distribution included four patients (40%) with grade 1, two (20%) with grade 2, and four (40%) with grade 3. Of these, three EC patients (EC2, EC4, and EC8) tested positive for the peritoneal fluid. The median follow-up period was 46 months (range: 29–62 months). Seven of the 10 patients showed no recurrence or progression; EC4 (stage III patients) showed a recurrence 7 months after the surgery and then reached No Evidence of Disease (NED); EC8 went through progression 5 months after the surgery and then reached NED; and EC9 (stage IV) showed progression without NED. All the patients underwent adjuvant treatment (chemotherapy and radiotherapy).

According to the ESGO (European Society of Gynaecological Oncology) 2016 risk stratification criteria, patient EC3 had a high-intermediate risk, eight patients were at high risk, and EC9 was metastatic. The ESGO 2021 criteria were assessed and are reported in Table 2 [6,40]. After applying the ESGO 2021 molecular classification, the risk group of one EC patient (EC6) changed from ‘high-risk’ to ‘high-intermediate risk’ as a result of the MSI status.

### 3.2. Mutation Profiles of the Peritoneal Fluid ctDNA, Plasma ctDNA, and Tumor gDNA Derived from EC Patients

**Figure 2:**
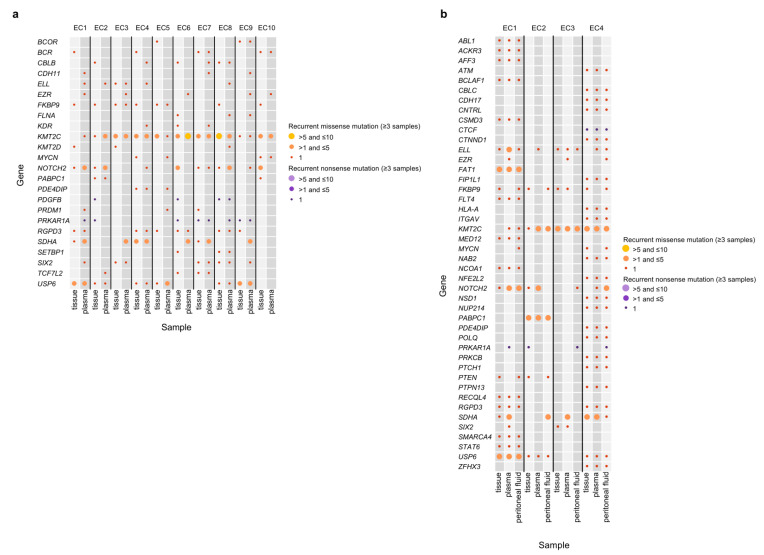
Recurrent somatic mutations (≥3 samples) in matched tissue and ctDNA samples using 401 cancer-related genes. (**a**) Tissue and plasma samples. (**b**) Tissue, plasma, and peritoneal fluid samples. The size of each dot indicates the number of recurrences. The color of each dot shows the type of somatic mutations. Samples are shown on the *x*-axis, and genes are listed on the *y*-axis.

We analyzed 34 samples (10 tumor tissues, 10 plasma, 4 peritoneal fluid, and 10 matched buffy coat), which passed the quality check, from 10 patients with EC. The coverage of sequencing depth was at a median of 206.7X (174.6–226.4X) for tissues, 171.2X (141.6–216.5X) for the plasma, 199.0X (161.3–246.7X) for the peritoneal fluid, and 206.0X (165.3X–264.1X) for matched buffy coat samples (Table S1). The descriptions of gDNA and ctDNA are summarized in Table 3. We identified a total of 401 cancer-related genes from the Cancer Gene Census (http://cancer.sanger.ac.uk/census; accessed on 10 August 2021) and 20 genes that were previously reported in ECs [1,12,13,32,33,34]. Somatic mutations were analyzed in paired tissue, plasma, and peritoneal fluid samples from four patients with EC and paired tissue and plasma samples from 10 patients with EC (Figure 1c, Figure 2a,b, and Table S2).

First, mutant allele frequencies (MAFs) of mutations shared between the tissue and plasma, tissue and peritoneal fluid, and plasma and peritoneal fluid samples were investigated (Figure 1b). Using Spearman’s correlation analysis, we found significant positive correlation between tissue and peritoneal fluid samples with a coefficient value of r = 0.628 and *p*-value < 2.2 × 10^−16^ (Figure 1b). Moderate positive correlations were found between tissue and plasma samples and between plasma and peritoneal fluid samples (Figure 1b).

Consistent with the correlation analysis, we identified concordance of somatic mutations in the top 20 EC-related genes, based on the COSMIC database (https://cancer.sanger.ac.uk/cosmic; accessed on 15 August 2021), among peritoneal fluid ctDNA, plasma ctDNA, and tumor gDNA (Figure 1c). Notably, we found *TP53* and *POLE* in EC1, EC3, EC4, and EC7; mutations in these genes have been shown to contribute to the categorization of ECs into molecular subtypes in previous studies [1,36]. The TP53 non-silent mutations that were detected in four EC patients (EC1, EC3, EC4, and EC7) were detected in the tissues except EC4 (Table 2, Tables S2 and S3). Nonsense and missense mutations (p.R174X and p.R181C) in *TP53* were identified in both peritoneal fluid and plasma ctDNA samples of EC4 patient, but not in tissues (Figure 1c, Table 2 and Table S2).

One missense mutation (p.A465V) in *POLE*, which was previously reported to be associated with ECs, was identified in both the plasma and peritoneal fluid ctDNA of EC4 (Figure 1c and Table S2) [41]. This mutation has been classified as a *POLE* EDM mutation and is located in the exonuclease domain of *POLE*, which causes an unusually high mutational burden [42]. The presence of *POLE* EDM mutation is one of the criteria used to categorize ECs into molecular subtypes [1,36]. ctDNA from the plasma and peritoneal fluid of EC4 and plasma ctDNA from EC5 with *POLE* mutations showed a high tumor mutational burden (TMB) of ≥20 mutations per megabase [43].

In addition, *ARID1A* harbored four nonsense and two missense mutations in seven patients with EC. One nonsense mutation (p.Q537X) in *ARID1A* was identified in both the peritoneal fluid ctDNA and tumor gDNA of EC2 (Figure 1c). *PIK3CA*, which has been frequently reported to possess mutations in EC genomic studies, harbored five missense mutations in four EC patients [1,32,33]. One missense mutation (p.R38H) in *PIK3CA* was identified in both peritoneal fluid ctDNA and tumor gDNA in EC1 patient, and another missense mutation (p.R38C) in *PIK3CA* was identified in both the peritoneal fluid and plasma ctDNA of EC4 (Figure 1c).

Next, recurrent non-silent mutations (≥3 samples) in 401 cancer-related genes were analyzed in peritoneal fluid ctDNA, plasma ctDNA, and tumor gDNA samples (Figure 2a,b). We found recurrent non-silent mutations in 46 genes among the three DNA sources (tissues, peritoneal fluid, and plasma) and in 26 genes between plasma ctDNA and tumor gDNA. Remarkably, 23 of the 24 samples showed recurrent missense mutations in *KMT2C*. In terms of recurrent nonsense mutations, mutations in 10 of 24 samples were detected in *PRKAR1A*.

In addition, PolyPhen-2, PROVEAN, and SIFT analyses revealed that most putative driver mutations (672 of 1314, 51%) had damaging effects on protein function, as detected with at least two of the three methods employed (Table S3). Sixteen mutations in 11 genes were validated using Sanger sequencing (Figure S1).

### 3.3. Immunohistochemistry

**Figure 3:**
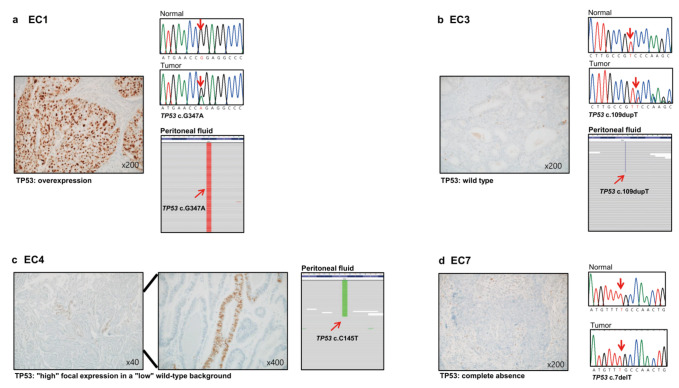
P53 immunohistochemical staining (left) and corresponding *TP53* mutations using sanger sequencing (tumor tissues) and whole-exome sequencing snapshot (peritoneal fluid) (right) (tissues; EC1, EC3, and EC7, peritoneal fluid; EC1, EC3, and EC4). (**a**) Left: cancer cells show diffuse strong nuclear staining in almost every nucleus. Right: validation of *TP53* mutation using Sanger sequencing of tissues and whole-exome sequencing snapshot from the peritoneal fluid (EC1). (**b**) Left: the tumor cells show wild type pattern. Right: validation of *TP53* mutation using Sanger sequencing of the tissue sample and whole-exome sequencing snapshot of the peritoneal fluid sample (EC7). (**c**) Left: tumor cells show a wild type pattern with a “high” focal expression in a “low” wild type background. Right: whole-exome sequencing snapshot of the peritoneal fluid sample (EC4). (**d**) Left: tumor cells are completely negative in all tumor cell nuclei. Right: validation of *TP53* mutation using Sanger sequencing of the tissue sample.

Immunohistochemistry for P53 was performed in all 10 EC tissues, and three EC tissues showed abnormal P53 expression levels (EC1, EC4, and EC7) (Table 2). EC1 showed overexpression of P53, EC4 showed a wild type pattern with a “high” focal expression in a “low” wild type background, and EC7 showed a complete absence (Figure 3a,c,d). Notably, EC4 harbored *TP53* mutations in the peritoneal fluid and plasma ctDNA, but not in the tissue (Table 2 and Table S2), indicating tumor heterogeneity.

### 3.4. Copy Number Alterations

**Figure 4:**
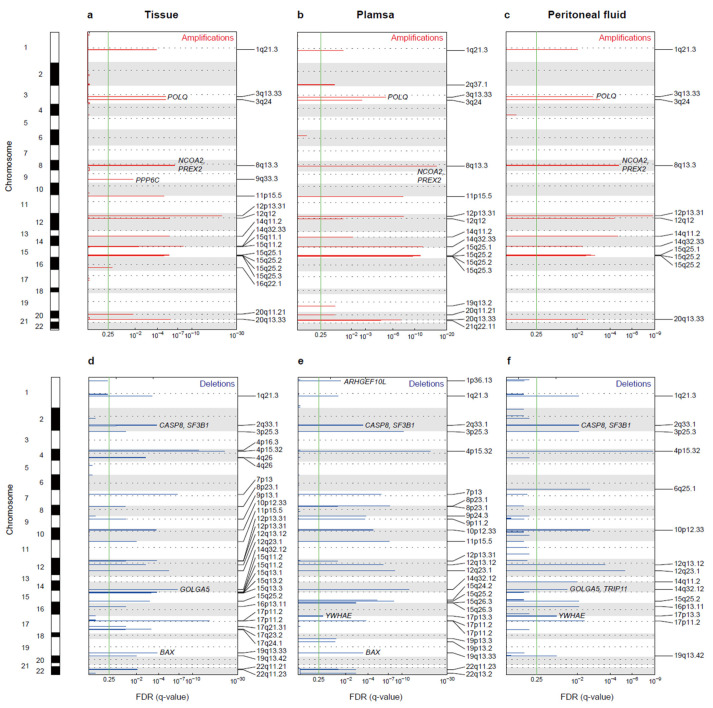
Recurrent CNAs in EC. (**a–c**) GISTIC2.0 analysis showing recurrent focal amplified regions and (**d**–**f**) deleted regions in the tissue, plasma, and peritoneal fluid samples of EC patients. The *x*-axis represents the FDR *q*-value, and the *y*-axis represents the chromosome. Cancer-related genes are labeled in each peak. Red and blue lines indicate amplification and deletion peaks, respectively. The green line indicates a *q*-value threshold of 0.25.

A total of 650 CNAs (254 gains and 396 losses) were identified in tissue, plasma, and peritoneal fluid samples from 10 patients with EC (Table S4). We found 52 recurrently altered CNA regions in tissues, 46 in plasma, and 27 in peritoneal fluid. Losses at 4p15.32 (tissue, plasma, and peritoneal fluid) and gains at 12p13.31 (tissue and peritoneal fluid) were the most recurrent events in ECs (Figure 4a–f and Table 3). In terms of cancer-related genes, *POLQ* and *PREX2* in the regions of gains and *CASP8* and *SF3B1* in the regions of loss were found in all samples, including the tissue, plasma, and peritoneal fluid (Figure 4a–f).

Noticeably, altered CNA regions in *PREX2* and *SOX17* were found among the three DNA sources in the EC4 patient, who showed a recurrence of the disease after 15 months (CN gains at 8q11.1–8q24.3 in the tissue, CN gains at 8q13.2–8q13.3 in the plasma, and CN gains at 8q11.1–8q13.2 and 8q13.2–8q13.3 in the peritoneal fluid) (Figure S2a,b). Common CN loss regions at 2p24.3–2p24.2 and 2q32.3–2q33.1 in *MYCN*, *SF3B1*, and *CASP8* were identified in both tissue and peritoneal fluid samples from the EC1 patient (Figure S2c). In addition, the CNA profiles of the EC7 patient were largely in agreement with previously identified CNAs in EC-related genes, such as *CDH1, CTNNB1, KRAS, MYC,* and *PIK3CA* (Figure S2d) [1,32,33]. Two main clusters were identified via hierarchical clustering of CNAs in the tumor gDNA samples (Figure S3a). Most endometrioid ECs in TCGA showed low copy number alterations, and the cluster in EC7 resembled the high copy number cluster 4 in TCGA [1]. Among the tumor gDNA and ctDNA samples analyzed, 23 of 24 were identified as low copy number samples, and all patients were classified as having endometrioid EC (Table 2).

### 3.5. Microsatellite Instability

We identified the MSI status in all EC genomes, as it makes a crucial contribution to diagnosis and therapeutic decisions in EC [6]. MSI status was identified in both tumor gDNA and peritoneal fluid ctDNA in 75% of the samples, MSS in EC2, and MSI in EC1 and EC4 (Figure 1c). Similarly, we observed MSS in paired tumor gDNA and plasma ctDNA samples from five patients with EC (Figure 1c). Among the four classified molecular groups, the MSI status of tumor gDNA and peritoneal fluid ctDNA showed concordance in patients EC1 and EC4 (Figure S3b).

### 3.6. Mutational Signatures

**Figure 5:**
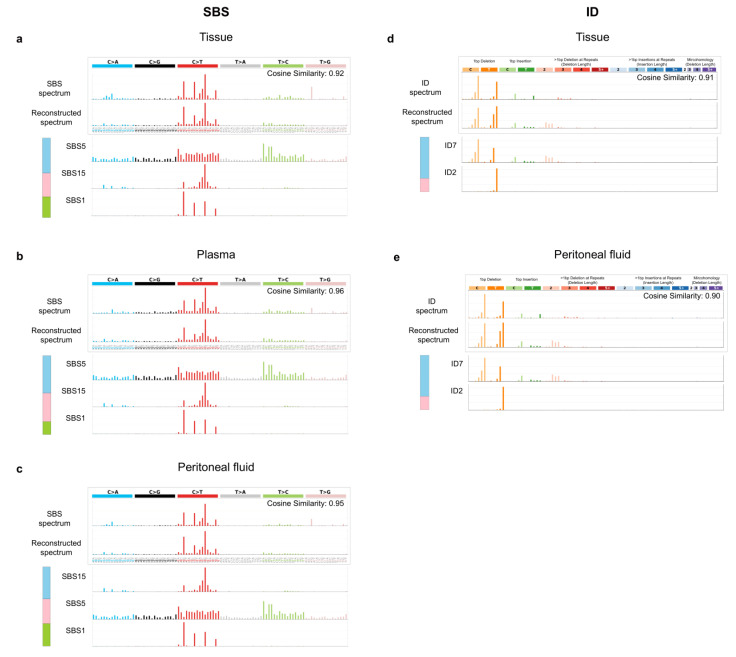
Mutational signatures in EC. (**a**–**c**) SBS and (**d**,**e**) ID from the COSMIC database identified in tissue, plasma, and peritoneal fluid of EC patients are indicated (cosine similarity ≥ 0.9). Bar plot indicates the proportion of each signature. Abbreviation: SBS, single-base-substitution; ID, small insertion-and-deletion.

Mutational signatures of somatic mutations were analyzed in peritoneal fluid ctDNA, plasma ctDNA, and tumor gDNA samples using the NMF algorithm. We found that C > T transitions, which are frequently found in *TP53* and human tumors with oxidative DNA-damaging effects, were abundant in all three DNA sources [44,45]. Mutational signatures SBS1, SBS5, and SBS15 in the COSMIC database were consistently detected in all three DNA sources with a cosine similarity above 0.9 (Figure 5a–c). It has been reported that all three SBS mutational signatures are frequently found in uterine or cervical cancers and that SBS15 is associated with defective DNA mismatch repair [26]. SBS15 is also one of the mutational signatures in the hypermutated (MSI) subtype of TCGA ECs [46]. SBS1 is related to endogenous mutational processes in most cancers [26]. Ageing-related SBS1 was the most frequently observed signature in ECs from TCGA, especially in the copy-number-low and copy-number-high groups [46]. Notably, we found the mutational signatures ID7 and ID2, which have been shown to be associated with DNA mismatch repair deficiency in cancers, in both tissue gDNA and peritoneal fluid ctDNA (cosine similarity of 0.91 and 0.90, respectively) (Figure 5d,e) [26].

### 3.7. Relation of Positive Peritoneal Fluid and Its Genetic Characteristics

According to the NCCN guidelines, peritoneal/ascitic fluid cytology is included in the principle of surgery, although cytology alone does not affect the FIGO staging (https://www.nccn.org; accessed on 20 September 2021). Peritoneal fluid was obtained from 10 patients with EC, and WES was performed on four samples (EC1, EC2, EC3, and EC4). Of these, EC1 and EC3 samples showed negative cytology results, while EC2 and EC4 displayed positive cytology (Table 1 and Table 2). As the number of patients was very small (only four EC patients), no statistically significant differences were observed in the mutation numbers of patients with negative (EC1 and EC3) and positive (EC2 and EC4) cytology. However, data showed that patients with negative cytology had higher total mutation numbers than those with positive cytology in both tissues (EC1: n = 2894, EC3: n = 2077 and EC2: n = 722, EC4 = 1472) and peritoneal fluid (EC1: n = 4044, EC3: n = 2169, and EC2: n = 1040, EC4 = 2911). We found that patients with negative peritoneal fluid (EC1 and EC3) harbored several important genetic features, such as two frameshift deletions (p.K250fs and p.P20fs), one missense mutation (p.R248Q), and one frameshift insertion (p.S37fs) in *TP53* and MSI. Patients EC1 and EC3 harbored concordant *TP53* mutations in tissues and the peritoneal fluid, and EC1 presented MSI-H status in both the tissue and peritoneal fluid. In addition, patients with positive peritoneal fluid (EC4) presented with important genetic features such as *TP53* mutation and MSI-H status.

## 4. Discussion

The mutational concordance of gDNA and ctDNA has only been partially supported by previous findings in EC genomes owing to the limited use of technology [12,13]. In this study, the comprehensive genomic profiles of ECs were investigated to determine the clinical utility of ctDNA from peritoneal fluid and plasma samples using WES and p53 immunohistochemistry. This study had two aims. First, it focused on identifying the genomic alteration profiles of ECs (somatic mutations, CNA, microsatellite instability, and mutational signatures). Second, it aimed to reveal the concordance between the peritoneal fluid, plasma, and tissue for the potential use of cfDNA as a noninvasive biomarker. Our data showed that genetic analysis of liquid biopsy samples (peritoneal fluid and plasma) could be performed to complement tissue heterogeneity. Next, we found that EC patients with negative peritoneal fluid harbored important genetic features similar to those of patients with positive peritoneal fluid. Finally, tissues, the peritoneal fluid, and the plasma presented reliable mutational correlations with respect to their MAF, genetic characteristics, and mutational signatures, although these parameters were not the same among the three types of clinical samples.

It was found that liquid biopsy using peritoneal fluid and plasma ctDNA can be performed to complement tumor heterogeneity. EC4 showed disease recurrence 15 months after surgery, and tissue gDNA showed a *TP*53wt phenotype. However, when p53 immunohistochemistry was performed, we found that EC4 presented a wild type pattern with a “high” focal expression in a “low” wild type background (Figure 3c). Abrupt and complete regional aberrant p53 expression of > 10% is typically defined as the ‘subclonal’ region. However, EC4 tissue harbored a regional aberrant p53 expression of < 10%, which was not sufficient to be characterized as “subclonal” [47]. Although the EC4 patient did not harbor *TP53* mutations in the tissue, *TP53* mutations were detected in both the peritoneal fluid and plasma. This may explain the ‘heterogeneity’ of P53 that was observed with immunohistochemistry. Molecular characterization revealed that patient EC4 had a ‘multiple classifier EC’ that harbored *POLE*mut (peritoneal fluid, plasma), *TP53*mut (peritoneal fluid, plasma), and MMRd (tissue, peritoneal fluid) (Table 2). These types of ‘multiple classifier ECs’ are very rare (0.3%), and very little is known about their prognosis; accordingly, further studies are needed [48].

The data presented the NCCN guidelines, including peritoneal/ascitic fluid cytology, as a principle of surgery, although positive peritoneal fluid does not affect the prognosis. Although it has been suggested that molecular characterization of peritoneal fluid is important [49], studies that have performed molecular characterization are limited in number. WES was performed using the peritoneal fluid ctDNA of patients with EC (4/10 patients). Of the four EC patients, two showed positive peritoneal fluid (EC2 and EC4) and the other two had negative peritoneal fluid (EC1 and EC3). Despite the absence of cancer cells from the peritoneal fluid in EC patients with negative peritoneal fluid, non-silent *TP53* mutations were found (Table 1 and Table 2, Figure 3a,b). In addition, ctDNA from the peritoneal fluid of patient EC1 presented a similar MSI-H status as tissue gDNA. These data suggest that molecular characterization of the peritoneal fluid, and not the findings of malignant cells in the peritoneal fluid, is important for the stratification of EC.

This comprehensive study showed that the ctDNA from liquid biopsy samples (peritoneal fluid and plasma) harbors important genetic biomarkers, and substantial correlations exist among them. We found that ECs harbored not only known mutations in driver genes, such as *ARID1A*, *CTNNB1*, *PIK3CA*, and *PTEN*, but also novel mutations in cancer-related genes, such as *KMT2C* and *PRKAR1A*. Notably, there was strong mutational concordance between the gDNA from tissues and ctDNA from the plasma and peritoneal fluid in well-known cancer-related genes (*ARID1A*, *PIK3CA*, *KMT2C*, and *PRKAR1A*, *TP53*). Interestingly, *KMT2C* was the most frequently mutated gene among the three DNA sources in our study; this gene has been previously reported in EC as a chromatin remodeling-related gene [50]. In *KMT2C*, at least one of the same recurrent missense mutations was found in all samples (Figure 2a). Additional recurrently mutated genes, *NOTCH2*, *PRKAR1A*, *USP6*, and *SDHA*, were also found in ECs with either missense or nonsense mutations.

These findings suggest that chromosomal alteration information obtained using ctDNA from the peritoneal fluid and plasma could provide a valuable tool for identifying EC. Most recurrent CNAs of ECs in the current study were largely in agreement with those described in earlier EC genomic studies, gains on 8q24.21, 8q11.2, 12p12.1, and 3q26–27 and losses on 4p16.3 and 3p14.2 from tissue samples [1,51]. To date, no EC studies have reported recurrent CNAs in peritoneal fluid or plasma samples. However, using WES, we found losses on 4p15.32 (tissue, plasma, and peritoneal fluid) and gains on 12p13.31 (tissue and peritoneal fluid) as the most recurrent events in EC. In terms of cancer-related genes, novel recurrent CNAs were identified in *PREX2* (8q gain) and *POLQ* (3q gain) in the peritoneal fluid, plasma, and tissue. Frequent losses on 2q33.1 in *CASP8* from the peritoneal fluid, plasma, and tissue have not been reported in EC; however, they have been reported to be highly related to cell death and drug resistance [52].

MSI or MMR status was detected using mutational signature analysis and MSI analysis of tissues, the peritoneal fluid, and the plasma. Data showed that the majority of EC patients had the same MSI status in both tumor gDNA and ctDNA from liquid biopsy samples (tumor gDNA and peritoneal fluid ctDNA (75% concordance); tumor gDNA and plasma ctDNA (50% concordance)). The mutational signatures SBS1, SBS5, and SBS15 have been found in uterine and cervical cancers, of which SBS15 is initiated by defective DNA mismatch repair [26]. In addition, ID2 and ID7 signatures were detected with high cosine similarity in tissue and peritoneal fluid DNA sources from EC patients, and these signatures are known to be associated with defective DNA mismatch repair; ID2 indels are caused by slippage at poly T repeats during DNA replication [26]. MMR has been reported to occur in microsatellites that identify and fix incorrect indels that may occur during DNA replication and recombination [53]. MSI status in EC is crucial for diagnostic and therapeutic purposes. MSI status can be used to diagnose the type of EC, predict the risk of Lynch syndrome, identify prognosis, and predict the potential utility of immune checkpoint inhibitors and targeted therapies [6,54].

Among the four classified molecular subtypes of EC genomes, several interesting observations were made (Figure S3b). TCGA showed that ECs with *POLE* mutations exhibited a high mutational burden in tumor gDNA, and a consensus in ctDNA was further identified by investigating all samples with *POLE* mutations [1]. In addition, TCGA classified the copy-number low group as an endometrioid group, as most endometrioid ECs have few copy number alterations [1]. All EC patients in the study had endometrioid ECs, and 23 of 24 samples, including gDNA and ctDNA, were classified into copy-number-low groups. These results demonstrate that the subtyping results for tumor gDNA and ctDNA were consistent with TCGA results. Although the sample size used was relatively small compared with that in other WES studies using population-based samples, this is the first study to provide comprehensive genomic information on ctDNA from ascites and plasma as non-invasive biomarkers in EC using WES.

## 5. Conclusions

To the best of our knowledge, this is the first study to compare comprehensive genomic profiles of ctDNA from the peritoneal fluid, plasma ctDNA, and tumor gDNA samples in EC genomes at the whole-exome level. The data provide clues that ctDNA can be used clinically to identify the mutational landscape of endometrial cancer and complement tissue heterogeneity.

## Figures and Tables

**Table 1:** Clinical information of the 10 patients with endometrial cancer.

ID	Pathologic Diagnosis	Age (yr)	FIGO Stage (2009)	Histologic Grade	Tumor Size (cm)	LVSI	Peritoneal Fluid	Recurrence/Progression	Current Status	Follow Up Period (mo)	Adjuvant Treatment	Others
EC1	Endometroid adenocarcinoma	62	IIIA	G3	7	(−)	(−)		NED	62	Chemotherapy and radiotherapy	
EC2	Endometroid adenocarcinoma	50	IIIC1	G1	8	(+)	(+)		NED	53	Chemotherapy and radiotherapy	
EC3	Endometroid adenocarcinoma	74	IB	G1	3	(+)	(−)		NED	47	Radiotherapy	
EC4	Endometroid adenocarcinoma	55	IIIC2	G3	10	(−)	(+)	Recur 15 mo after the surgery.	NED	47	Chemotherapy and radiotherapy	
EC5	Endometroid adenocarcinoma	45	II	G1	9	(+)	(−)		NED	47	Radiotherapy	
EC6	Endometroid adenocarcinoma	54	IB	G3	5	(+)	(−)		NED	45	Radiotherapy	
EC7	Endometroid adenocarcinoma	67	IIIC1	G3	4	(+)	(−)		NED	42	Chemotherapy and radiotherapy	
EC8	Endometroid adenocarcinoma	74	IIIC1	G2	6	(+)	(+)	Progression 5 mo after the surgery.	NED	39	Chemotherapy and radiotherapy	
EC9	Endometroid adenocarcinoma	59	IVB	G1	7	(+)	(−)	Never NED	Under treatment	33	Chemotherapy and radiotherapy	Incomplete surgical resection and lung metastasis
EC10	Endometroid adenocarcinoma	51	IIIC2	G2	12	(+)	(−)		NED	29	Chemotherapy, radiotherapy	

Abbreviation: NED: no evidence of disease; LVSI, lymphovasculostromal invasion.

**Table 2:** Molecular classification of the 10 patients with endometrial cancer.

		EC1	EC2	EC3	EC4	EC5	EC6	EC7	EC8	EC9	EC10
*P53* mut **	Tissue	mut	wt	mut	wt	wt	wt	mut	wt	wt	wt
	Plasma	wt	wt	wt	mut	wt	wt	wt	wt	wt	wt
	Peritoneal fluid *	mut	wt	mut	mut						
TP53 immunohisto-chemistry	Tissue	Overexpression	wt	wt	wt ***	wt	wt	Complete absence	wt	wt	wt
POLE mut **	Tissue	wt	wt	wt	wt	wt	wt	wt	wt	wt	wt
	Plasma	wt	mut	wt	EDM	mut	wt	wt	wt	wt	wt
	Peritoneal fluid *	wt	wt	wt	EDM						
MSI status	Tissue	MSI-H	MSS	MSI-H	MSI-H	MSS	MSI-H	MSS	MSS	MSS	MSI-H
	Plasma	MSS	MSS	MSS	MSS	MSS	MSS	MSS	MSS	MSS	MSS
	Peritoneal fluid *	MSI-H	MSS	MSS	MSI-H						
Copy number low/ high	Tissue	low	low	low	low	low	high	low	low	low	low
	Plasma	low	low	low	low	low	low	low	low	low	low
	Peritoneal fluid *	low	low	low	low						
Risk group (ESGO 2016)	Tissue	High	High	High-intermediate	High	High	High	High	High	Metastatic	High
Risk group (ESGO 2021), molecular classification known	Tissue	High	High	High-intermediate	High	High	High-intermediate	High	High	Metastatic	High

* Molecular evaluation of the peritoneal fluid was performed only for EC1, EC2, EC3, and EC4; ** The mutations detected in one EC patient were shared among tissue, plasma, and peritoneal fluid; *** It is a wild type pattern with focal “high” expression in “low” wild type background; EDM, endonuclease domain mutation; ESGO, European Society of Gynaecological Oncology; MSI-H, microsatellite instability-high (~MMR deficient); MSS, microsatellite stable; mut, mutation; NA, not applicable; wt, wild type.

**Table 3:** Summary of comparison of the data from tissue, plasma, and peritoneal fluid samples for the EC genomes.

	Tissue (N = 10)	Plasma (N = 10)	Peritoneal Fluid (N = 4)
Somatic mutation number	7908.6/tumor	8127.1/tumor	12,241.3/tumor
Mutation allele frequency	0.05/variant	0.20/variant	0.07/variant
Nonsilent mutation number	8087.3/tumor	7546.3/tumor	12,526/tumor
Mutation allele frequency (nonsilent)	0.07/variant	0.19/variant	0.09/variant
Silent mutation number	861.2/tumor	1150.4/tumor	1517/tumor
Mutation allele frequency (silent)	0.03/variant	0.24/variant	0.04/variant
Putative driver mutation number	72.2/tumor	99.7/tumor	154.75/tumor
Representative driver mutation	*FKBP9*, *KMT2C*, *USP6*	*KMT2C*, *NOTCH2*, *SDHA*, *USP6*	*KMT2C*, *NOTCH2*, *USP6*
No. of CNAs	290 (median: 27.5)	232 (median: 23.0)	128 (median: 32.5)
Length of CNAs (Mb)	2046.8 (median: 106.1)	568.3 (median: 50.9)	337.6 (median: 62.4)
Most recurrent copy gain	12p	8q	12p
Most recurrent copy loss	4p	4p	4p
MSI	MSI (n = 5)	MSI (n = 0)	MSI (n = 2)

Abbreviation: CNAs, copy number alterations; MSI, microsatellite instability; “Somatic mutation number” is the total number of somatic mutations, thus we only count one alternative allele from each multiallelic SNP.

## Data Availability

The data presented in this study are available upon request from the corresponding author.

## Supplementary Materials

The following supporting information can be downloaded at: https://www.mdpi.com/article/10.3390/cancers14102506/s1, Figure S1: Validation of mutations using Sanger sequencing; Figure S2: CNA results of tissue, plasma, and peritoneal fluid samples in three EC patients; Figure S3: Samples were hierarchically clustered based on CNAs and categorized into four groups according to MSI status, *POLE* EDM, and copy number cluster; Table S1: Description of whole-exome sequencing data of endometrial carcinoma genomes; Table S2: Somatic point mutations and indels identified in the endometrial cancer genomes.; Table S3: Predicting the functional effects of somatic mutations in endometrial cancer genomes; Table S4: Copy number alterations identified across 24 endometrial cancer genomes using WES.
