# Supplementary material for: Whole-Exome Sequencing Reveals Clinical Potential of Circulating Tumor DNA from Peritoneal Fluid and Plasma in Endometrial Cancer
Source: Cancers (Basel). 2022 May 19;14(10):2506. doi: 10.3390/cancers14102506 (PMC9139435; doi:10.3390/cancers14102506)
Supplement: Supplementary file 1 [file cancers-14-02506-s001.zip › Supplementary_Figures.pdf]

Figure S1. Validation of mutations using Sanger sequencing.

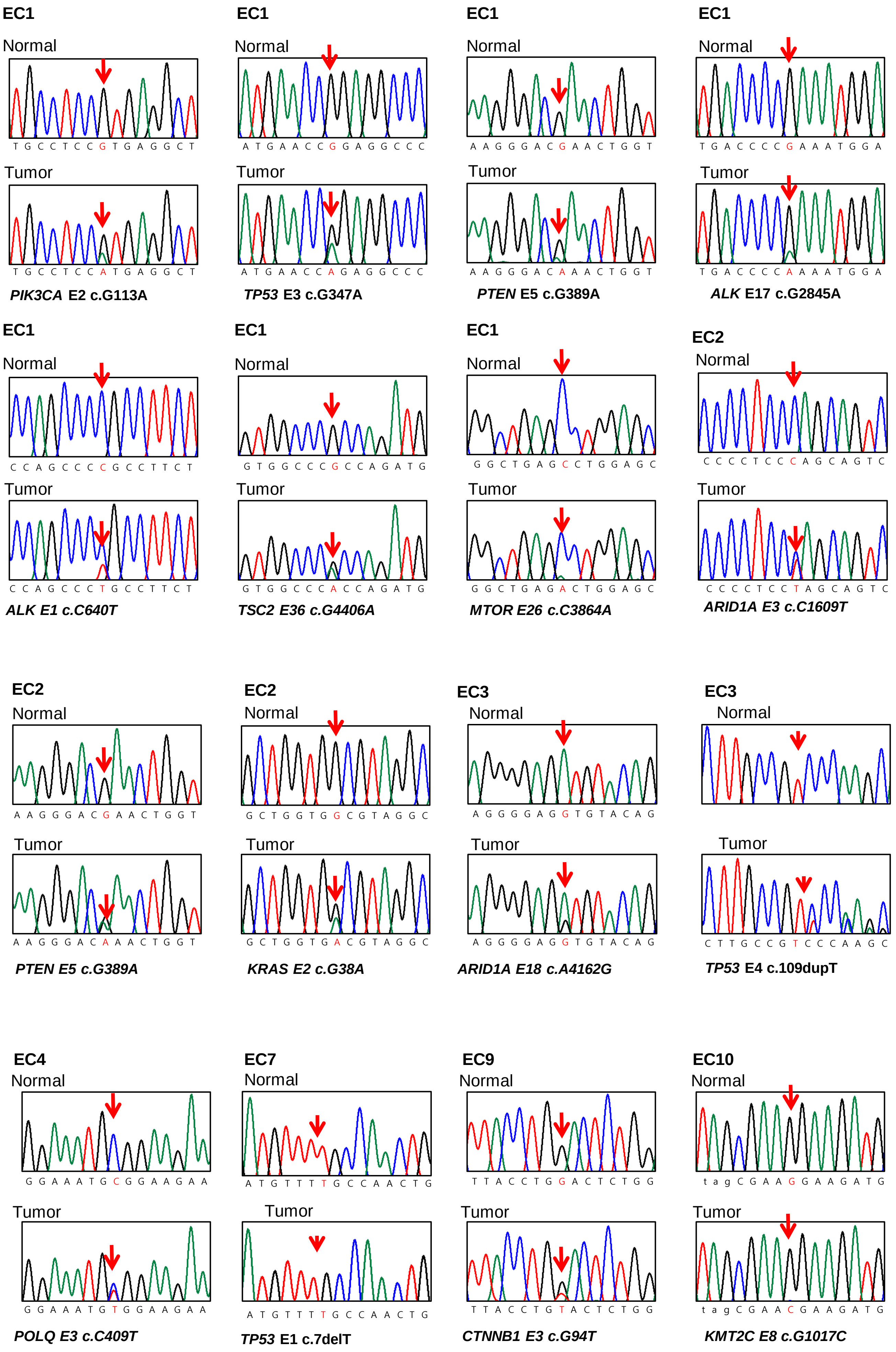

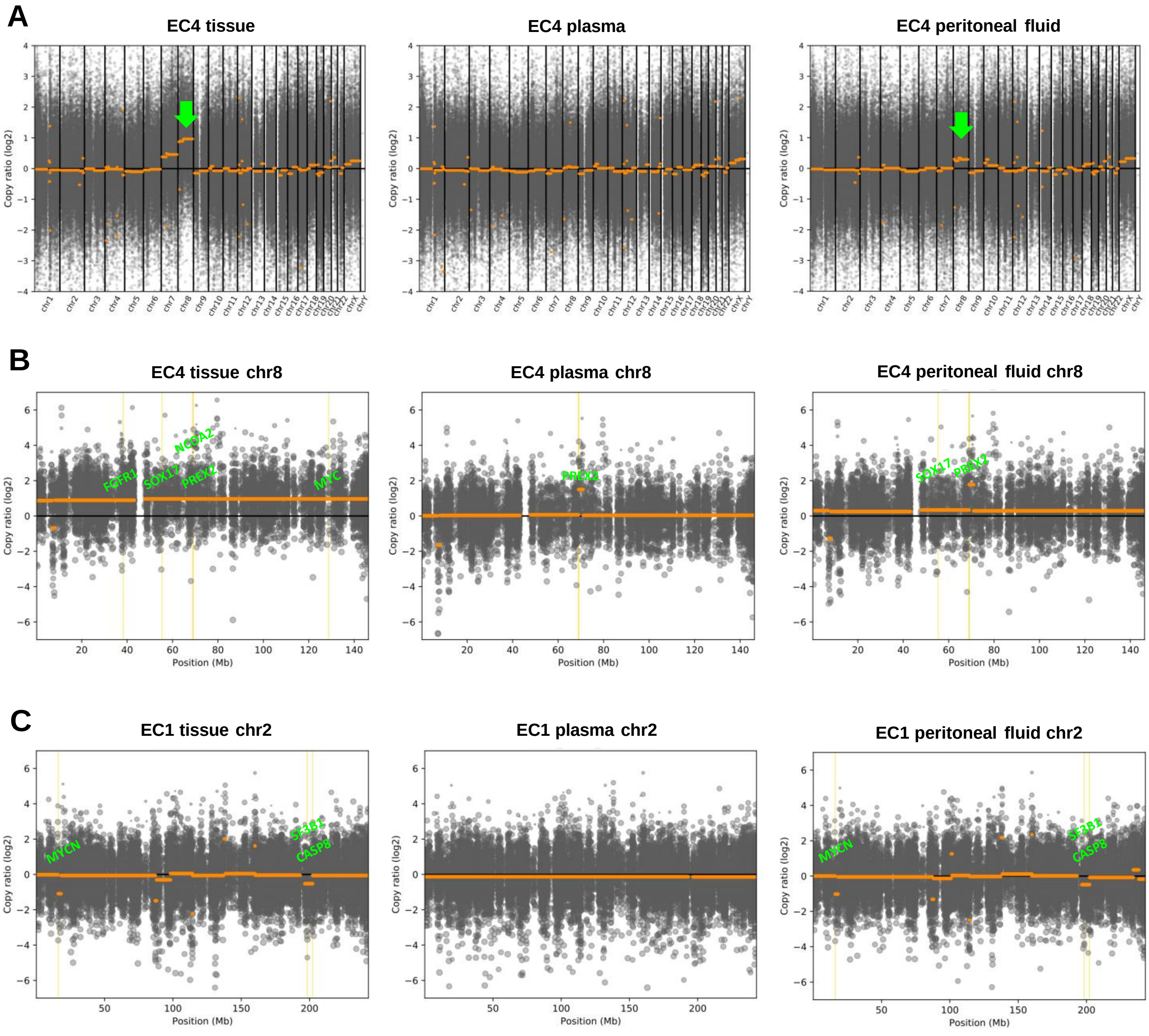

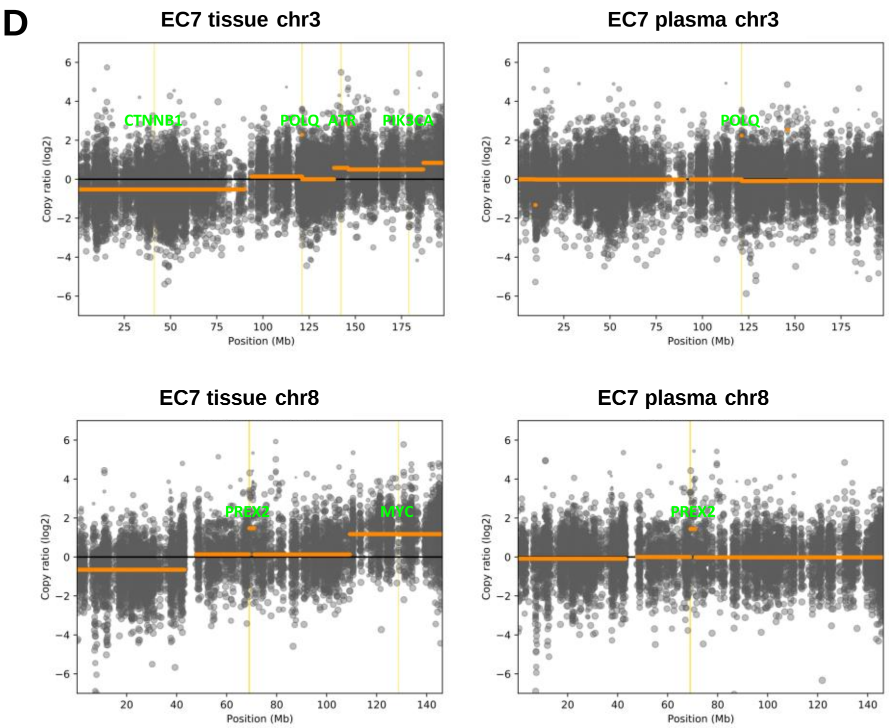

Figure S2. CNA results of tissue, plasma, and peritoneal fluid samples in three EC patients. X-axis indicates position and y-axis indicates log2 copy number ratio. (A) CNA of entire chromosomal region. (B) CNA of chromosome 8 in EC4. Genes associated with EC are shown. (C) CNA of chromosome 2 in EC1. (D) CNA of paired tissue and plasma samples in EC7.

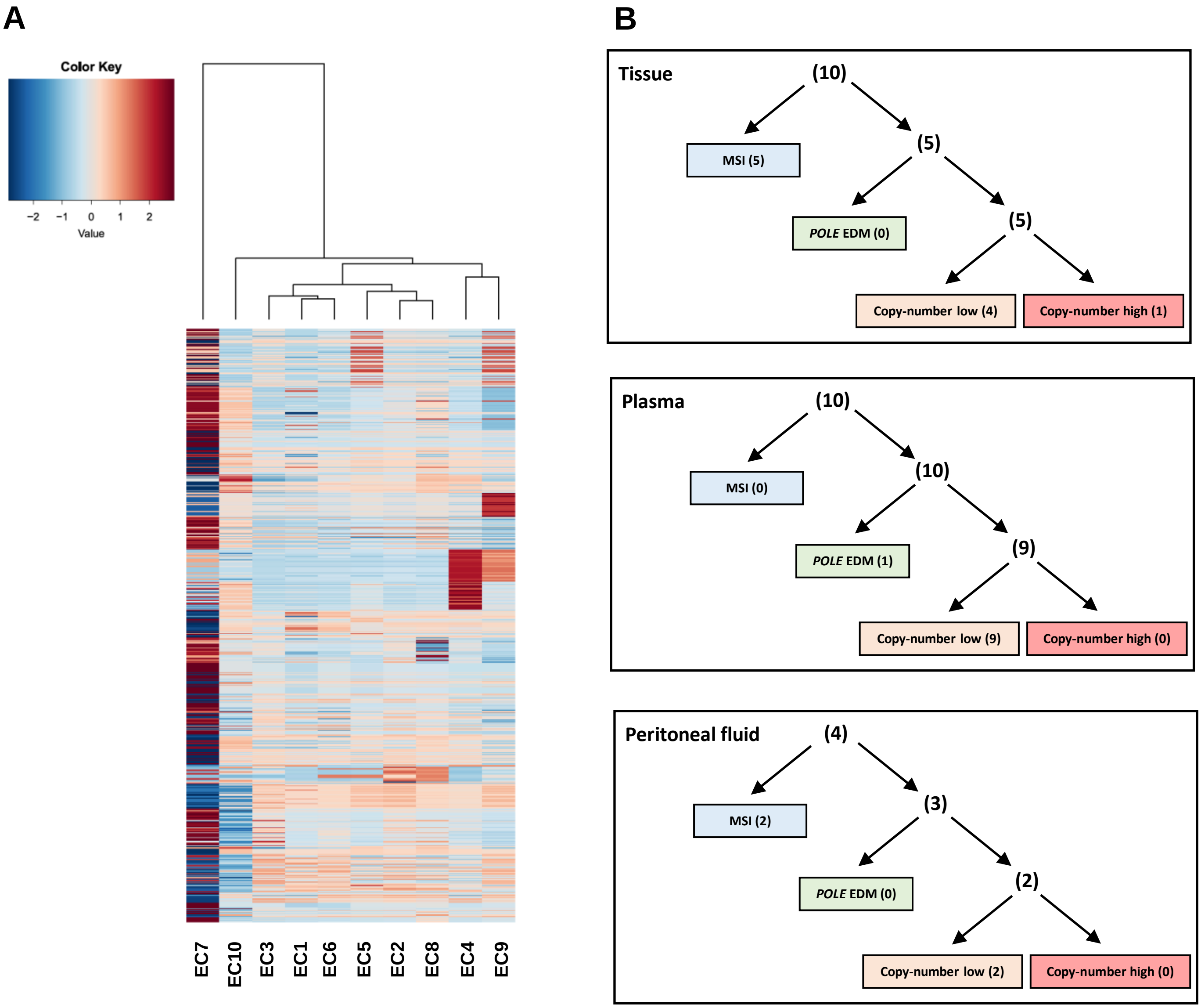

Figure S3. Samples were hierarchically clustered based on CNAs and categorized into four groups according to MSI status, *POLE* EDM, and copy-number cluster. (A) Heatmap showing CNAs in each tumor gDNA (horizontal axis) plotted with respect to cancer-related genes from the Cancer Gene Census and genes previously reported in EC (vertical axis). (B) Samples classified into four groups are shown in tumor gDNA, plasma ctDNA, and peritoneal fluid ctDNA, respectively.
